# Polymodal Functionality of *C. elegans* OLL Neurons in Mechanosensation and Thermosensation

**DOI:** 10.1007/s12264-021-00629-4

**Published:** 2021-02-08

**Authors:** Yuedan Fan, Wenjuan Zou, Jia Liu, Umar Al-Sheikh, Hankui Cheng, Duo Duan, Du Chen, Siyan Liu, Luyi Chen, Jilei Xu, Firdosh Ruhomutally, Lijun Kang

**Affiliations:** 1 Department of Neurobiology and Department of Neurosurgery of the First Affiliated Hospital, Zhejiang University School of Medicine, Hangzhou, 310053 China; 2 NHC and CAMS Key Laboratory of Medical Neurobiology, MOE Frontier Science Center for Brain Research and Brain-Machine Integration, School of Brain Science and Brain Medicine, Zhejiang University, Hangzhou, 310053 China; 3 Department of Gastroenterology, Sir Run Run Shaw Hospital, Zhejiang University School of Medicine, Hangzhou, 310016 China; 4 Department of Human Sciences and Psychology, University of South Africa (UNISA), Pretoria, 0003 South Africa

**Keywords:** Polymodal sensory neuron, Mechanosensation, Thermosensation, Sodium channel, Cold receptor, OLL neurons

## Abstract

Sensory modalities are important for survival but the molecular mechanisms remain challenging due to the polymodal functionality of sensory neurons. Here, we report the *C. elegans* outer labial lateral (OLL) sensilla sensory neurons respond to touch and cold. Mechanosensation of OLL neurons resulted in cell-autonomous mechanically-evoked Ca^2+^ transients and rapidly-adapting mechanoreceptor currents with a very short latency. Mechanotransduction of OLL neurons might be carried by a novel Na^+^ conductance channel, which is insensitive to amiloride. The *bona fide* mechano-gated Na^+^-selective degenerin/epithelial Na^+^ channels, TRP-4, TMC, and Piezo proteins are not involved in this mechanosensation. Interestingly, OLL neurons also mediated cold but not warm responses in a cell-autonomous manner. We further showed that the cold response of OLL neurons is not mediated by the cold receptor TRPA-1 or the temperature-sensitive glutamate receptor GLR-3. Thus, we propose the polymodal functionality of OLL neurons in mechanosensation and cold sensation.

## Introduction

The ability to detect sensory cues is innate to living organisms to produce appropriate behavioral and physiological responses for survival [1]. Specialized neurons are often polymodal, responding to ambient stimuli in the form of chemicals, temperature, and touch intensity [1, 2]. In mammals, nociceptive neurons located in the dorsal root ganglion and trigeminal ganglion are activated by noxious chemicals, extreme temperatures, and mechanical stimuli [3]. *Drosophila* class IV da neurons are well-known polymodal neurons, which can detect diverse forms of stimuli, such as thermal, mechanical, proprioceptive, and light stimuli [4–7]. The nematode *Caenorhabditis elegans* stands as an excellent model for dissecting sensory mechanisms with its 302 confirmed hermaphrodite neurons, and numerous behavioral analyses can be readily performed in the small and rapidly-growing worm. Neurons in *C. elegans* are often multifunctional, involved in complex behaviors through neuronal circuits and signaling molecules [1, 8–10].

The *C. elegans* outer labial lateral (OLL) sensilla sensory neurons negatively regulate pathogen avoidance behavior *via* HECW-1 to undergo adaptive changes in response to microbial pathogens. Laser ablation of OLL neurons enhances pathogen-avoidance behavior, suggesting that they play an important role in conserved innate immune responses [11, 12]. In addition, nose touch-defects were also reported from animals in which OLL was ablated genetically [11]. However, the nature of OLL neurons’ responses to environmental stimuli is yet to be determined at the cellular level, with the underlying molecular mechanisms still elusive.

In this study, we investigated the sensory responses of OLL neurons using miscellaneous stimuli, Ca^2+^ imaging, and electrophysiological recordings. By methodological micro-dissection of the nose tip, OLL neurons were exposed and responded to mechanical stimulation through a novel Na^+^ conductance channel. However, we ruled out degenerin/epithelial Na^+^ channel (DEG/ENaC) family proteins and found that this mysterious channel is amiloride-insensitive, and responsible for the mechanotransduction of OLL neurons in *C. elegans*. Apart from mechanotransduction, we also revealed that OLL neurons responded to cold stimulation cell-autonomously but not *via* the typical TRPA-1 channel [13, 14]. Therefore, we provide an avenue for the molecular dissection of sensory modalities in OLL neurons.

## Materials and Methods

### Strains

The *C. elegans* strains were raised on NGM plates seeded with OP50 bacteria at 20°C. We generated the transgenic strain kanEx1200[*Pser-2d::GCaMP6s *+ *Pser-2d:: mCherry* + *Plin-44::gfp*] by micro-injection, to detect intracellular Ca^2+^ activity in OLL neurons. The mutant strains used in this study were *unc-13(e51)*, *unc-31(e928)*, *trpa-1(ok999)*, *deg-1(u38)*, and *mec-10(e1515)*.

### Molecular Biology

The *ser-2d* promoter [15] was amplified by PCR using the primers 5′-acgctaacaacttggacatgcttgttctagtgatc-3′ and 5′-tttgggtcctttggccattatgtgttgtgatgtcacaa-3′. A 4.1-kb fragment generated from Bristol N2 genomic DNA was cloned into the PBS77 vector using In-fusion technology (Vazyme). We injected the N2 strain with *Pser-2d::GCaMP6s* at 80 ng/μL and *Pser-2d::mCherry* at 50 ng/μL, together with 20 ng/μL *Plin-44::gfp* as a co-injection marker.

### Calcium Imaging

Worms were glued to a glass-bottom cell culture dish covered with a thin layer of 5% agarose then immersed in bath solution. This solution consisted of (in mmol/L) 145 NaCl, 2.5 KCl, 5 CaCl_2_, 1 MgCl_2_, 20 glucose, adjusted to pH 7.3. We used an SC-20 Dual In-line Solution Heater/Cooler (Warner Instruments) to precisely regulate the temperature of the bath. Ca^2+^ imaging was carried out on an Olympus IX83 inverted microscope under a 40× objective with Olympus cellSens software. The temperature-stimulated Ca^2+^ imaging system was as described previously [16, 17].

### Touch-Stimulated Calcium Imaging

The touch-stimulated Ca^2+^ imaging protocol was as previously described [10, 16, 18]. Briefly, worms were glued on a coverslip and immersed in bath solution. Touch stimuli were delivered to OLL neurons using about 1 μm borosilicate glass capillary needle on a micromanipulator (Sutter). The needle was placed near the worm's nose and Patchmaster software (HEKA Elektronik) was used to control the micromanipulator. When executing the “touch” command, the needle moved forward and touched the worm’s nose for 500 ms then returned to the original position. Ca^2+^ imaging was recorded on an Olympus BX51WI upright microscope under a 60× objective with Macro-manager software. The bath solution consisted of (in mmol/L) 145 NaCl, 2.5 KCl, 5 CaCl_2_, 1 MgCl_2_, 20 glucose, adjusted to pH 7.3. In Na^+^-deprived bath solution, an equimolar concentration of N-methyl-D-glucamine chloride (NMDG-Cl) was substituted for NaCl.

### Electrophysiology

Whole-cell patch clamp recordings were carried on an Olympus BX51WI upright microscope under a 60× objective with an EPC-10 amplifier and Patchmaster software as previously described [19]. Briefly, a glass stimulus probe driven by a Piezo actuator was mounted on a micromanipulator and triggered by an amplifier. Mechanical stimulation continued for 500 ms. Worms were glued to a Sylgard-coated coverslip covered with bath solution, and a small piece of cuticle in the head was cut and pinned to the coverslip to expose the cell bodies of OLL neurons. To identify OLL neurons, the fluorescent protein marker mCherry-fused *ser-2d* plasmid was injected before dissection. Recording pipettes were pulled on a micropipette puller P-1000 (Sutter). The bath solution contained (in mmol/L) 145 NaCl, 2.5 KCl, 5 CaCl_2_, 1 MgCl_2_, and 20 glucose (325–335 mOsm, pH adjusted to 7.3). The intracellular solution consisted of (in mmol/L) 145 Cs-gluconate, 5 MgCl_2_, 5 EGTA, 0.25 CaCl_2_, 10 HEPES, 10 glucose, 5 Na_2_ATP, and 0.5 Na_2_GTP (315–325 mOsm, pH adjusted to 7.2). The membrane potential was clamped at −70 mV. The DEG/ENaC channel blocker amiloride was dissolved in the bath to a final concentration of 400 μmol/L.

### Primary Cell Culture

The protocol for *C. elegans* primary cell culture was as described previously [20]. In brief, day 2 adult (D2) worms were lysed to enrich the eggs (lysis solution: 5 mL fresh bleach, 1.25 mL 10 mol/L NaOH, and 18.5 mL sterile H_2_O). Eggs were digested with 2 mg/mL chitinase (Sigma, catalog no. C6137) and dissociated into single cells. Then the cells were implanted on glass-bottomed cell-culture dishes coated with peanut lectin (Sigma, catalog no. L0881) at a suitable cell concentration. The cells were cultured in L-15 medium (Invitrogen, catalog no. 21083-027) with 10% (v/v) heat-inactivated fetal bovine serum (Invitrogen, catalog no. 10082-139), 50 U/mL penicillin, and 50 mg/mL streptomycin (Invitrogen, catalog no. 15140-122). After two days in culture, Ca^2+^ imaging experiments were performed. The isolated OLL neurons were recognized by expression of *Pser-2d::mcherry* as well as *Pser-2d::GCaMP6s*. It has been reported that the posterior ventral dorsal (PVD) neurons sense cold *via* TRPA-1 channels and detect touch through ENaC channels [21, 22]. Given that PVD and OLL neurons share the same *ser-2d* promoter, we isolated cells of the *trpa-1* mutant strain for temperature-stimulated Ca^2+^ imaging to exclude PVD neurons. For touch-stimulated Ca^2+^ imaging in isolated OLL neurons, we immersed the cells in bath solution containing 400 μmol/L amiloride to rule out PVD neurons.

### Statistical Analysis

Data analysis was performed using ImageJ and GraphPad Prism 6. All data are presented as the mean ± SEM and unpaired two-tailed Student’s *t*-test was used to compare data sets. If the data were not normally distributed, the Wilcoxon test was used. *P* < 0.05 was considered to be statistically significant.

## Results

### OLL Neurons Respond to Touch and Cold

**Fig. 1:**
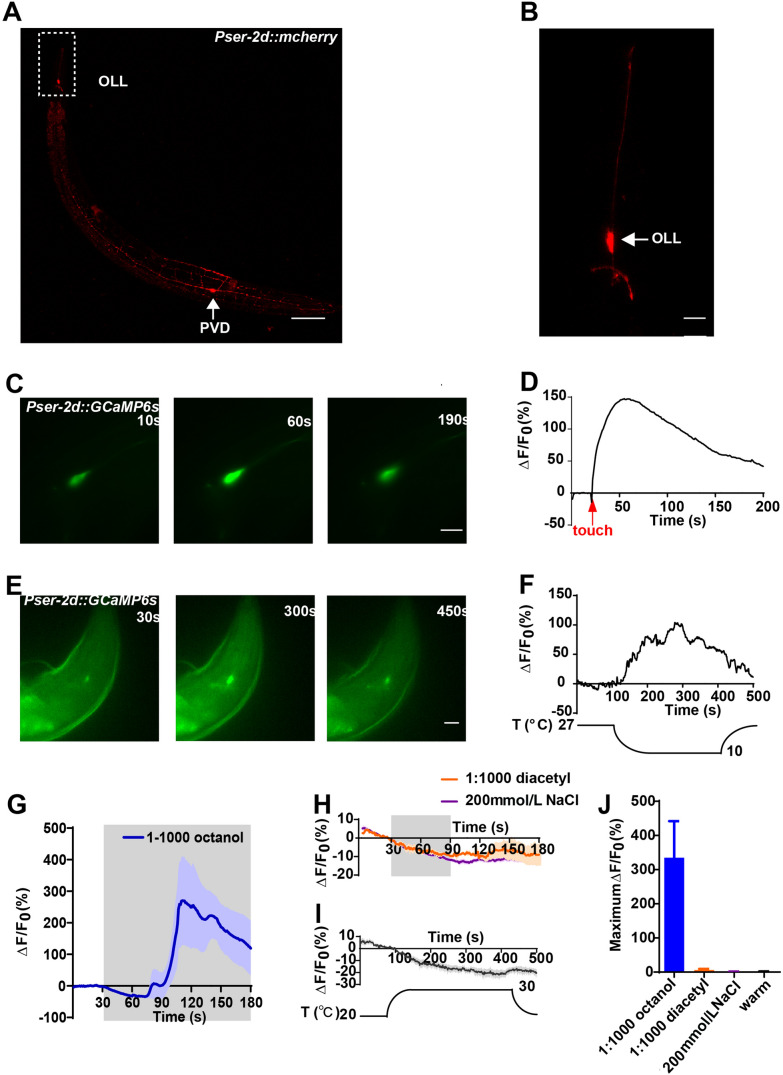
The response spectrum of *C. elegans* OLL neurons. **A** Expression pattern of *ser-2d* reporter gene fusion. *Pser-2d::mCherry* exclusively expresses in OLL neurons and PVD neurons (scale bar, 100 μm). **B** OLL labeled with *Pser-2d::mCherry* marker. **C, D** Representative time-lapse images of Ca^2+^ responses based on GCaMP6s (**C**) and soma fluorescence changes (**D**) from an OLL neuron in response to nose touch. A glass probe driven by a Piezo actuator is used to deliver a mechanical stimulus with a defined displacement towards the cilium of the OLL neuron. The stimulus displacement is 20 μm for 500 ms. Red arrow, time of mechanical stimulation. **E, F** Representative time-lapse images of Ca^2+^ responses based on GCaMP6s (**E**) and soma fluorescence changes (**F**) from an OLL neuron. The initial temperature of the recording buffer is 27°C, and the lowest temperature is 10°C. Cool-down is initiated at 100 s, then heated back to 27°C after 5 min (scale bars, 10 μm in **B, C, E**). **G** The repulsive odor 1-octanol (1:1000) induces Ca^2+^ responses in OLL neurons. Note that the delay of 1-octanol evoked Ca^2+^ transients is quite long (gray rectangle, duration of administration). **H** The attractive odor diacetyl (1:1000) and 200 mmol/L NaCl does not cause a Ca^2+^ response in OLL neurons (gray rectangle, duration of administration). **I** Warm stimulation-induced Ca^2+^ responses in OLL neurons. The initial temperature of the recording buffer is 20°C, and the highest temperature is 30°C. Heating is initiated at 100 s, then back to 20°C after 5 min. **J** Maximum ΔF/F_0_ changes (**D**) of odorants, salt, and warm stimulation-induced Ca^2+^ responses in OLL neurons.

OLL neurons (OLLL and OLLR) are neurons of the outer labial lateral sensilla with the axis cylinder processes extending to the tip of the worm’s nose (Fig. 1A, B). To explore the response spectrum of OLL neurons, we generated a transgenic strain expressing the Ca^2+^ indicator protein GCaMP6s and a red fluorescent protein mCherry under the control of the *ser-2d* promoter [15, 23]. The worms expressed GCaMP6s and mCherry in OLL neurons and another class of sensory neurons, the phasmid ventral cord (PVD) neurons (Fig. 1A). We then performed several sensory stimuli tests including the use of odorants, salt, warm and cold temperatures, and nose-touch that OLL neurons may detect. We found that mechanical stimulation evoked fast Ca^2+^ transients in OLL neurons (Fig. 1C, D). Furthermore, OLL neurons exhibited rapid and robust Ca^2+^ transients to cold stimulation when the buffer temperature was decreased from 27°C to 10°C (Fig. 1E, F). As such, OLL neurons respond to mechanical stimulation as well as cold stimulation. We also found that the repulsive odor 1-octanol [24, 25] (1/1000 in bath solution) induced Ca^2+^ increases in OLL neurons. However, the latency of these increases was extremely long (>30 s) (Fig. 1G, J), indicating that OLL neurons respond to 1-octanol in a non-cell-autonomous manner. Diacetyl (1/1000 in bath solution), which is attractive to worms [24, 26], did not evoke detectable Ca^2+^ transients in OLL neurons (Fig. 1H, J). In addition, high salt (200 mmol/L NaCl) and warm stimulation did not evoke Ca^2+^ responses in OLL neurons (Fig. 1H–J).

### The Amplitude of Calcium Responses in OLL Neurons Depends on the Strength of Mechanical Stimuli

**Fig. 2:**
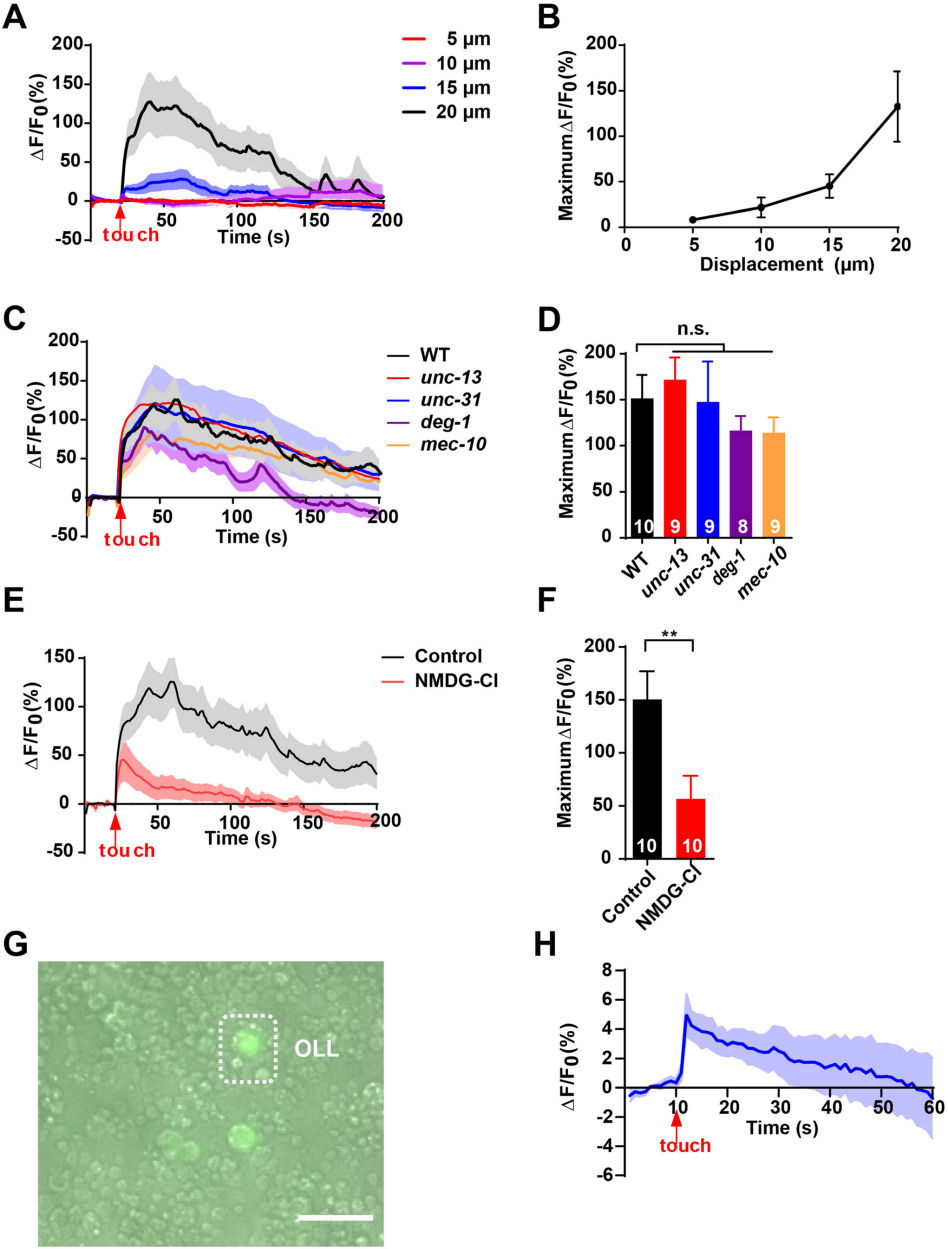
OLL neurons respond to mechanical stimulation in a cell-autonomous manner. **A**, **B** Ca^2+^ responses (**A**) and maximum ΔF/F_0_ changes (**B**) induced by mechanical stimulation. Displacements of 5 μm, 10 μm, 15 μm, and 20 μm are tested. Considering that harsh touch may cause irreversible deformation and damage, we did not use displacements >20 μm. The *n* values for the 5 μm, 10 μm, 15 μm, 20 μm displacement groups were 10, 10, 14, and 9, respectively. **C, D** Ca^2+^ responses (**C**) and maximum ΔF/F_0_ changes (**D**) induced by mechanical stimulation in OLL neurons of wild type (*n* = 10), *unc-13* mutants (*n* = 9), *unc-31* mutants (*n* = 9), *deg-1* mutants (*n* = 8), and *mec-10* mutants (*n* = 9). The stimulus displacement is 20 μm. **E, F** Ca^2+^ responses (**E**) and maximum ΔF/F_0_ changes (**F**) induced by mechanical stimulation. Worms of the experimental group are immersed in perfusate in which NMDG-Cl replaces NaCl. The stimulus displacement was 20 μm. *n* = 10 animals per condition. **G** Image of a culture of isolated *C. elegans* embryonic cells. Isolated OLL neurons are indicated by the expression of GCaMP6s (scale bar, 20 μm). **H** Mechanical stimulation-evoked Ca^2+^ responses in isolated OLL neurons. To rule out PVD neurons, we immersed the cells in 400 μmol/L amiloride. The stimulus displacement was 3 μm. *n* = 6. Error bars indicate SEM; n.s., not significant, *P* > 0.05; **P < 0.01.

To determine the threshold of mechanical stimulus-evoked Ca^2+^ responses in OLL neurons, we applied stimuli of varying intensities to the nose tip. We used a glass probe driven by a Piezo actuator to deliver a mechanical stimulus with a defined intensity to the cilium of OLL neurons. The results demonstrated that the increased touch-evoked Ca^2+^ responses depend on a significant increase of stimulus intensity, and a displacement of 15 μm was sufficient to induce a Ca^2+^ response (Fig. 2A, B). Consequently, the Ca^2+^ responses were rapid and robust when the stimulus displacement was up to 20 μm (Fig. 2A, B). Considering that harsh touch may cause irreversible deformation and damage, we refrained from displacements >20 μm. We then applied a stimulus of 20 μm displacement to the subsequent Ca^2+^ imaging experiments.

### OLL Neurons Sense Mechanical Stimulation Cell-Autonomously Through Sodium Channels

OLL neurons responded to mechanical stimulation rapidly and robustly (Fig. 2A), indicating that they function as primary sensory neurons for mechanosensation. To further verify this hypothesis, we measured their touch-induced Ca^2+^ responses in *unc-13* and *unc-31* mutants. The *unc-13* gene encodes UNC-13 protein, a Munc13 homolog in *C. elegans*, which is required for the release of neurotransmitters from synaptic vesicles [27, 28]. The *unc-31* gene encodes the mammalian ortholog CAPS in *C. elegans* that is essential for the release of neuropeptide from dense core vesicles [29]. The mechanically-evoked Ca^2+^ transients of OLL neurons in both *unc-13* and *unc-31* mutant worms did not differ from wild-type worms (Fig. 2C, D). These results support the hypothesis that OLL neurons detect mechanical stimulation cell-autonomously.

To further confirm that OLL neurons directly detect mechanical stimulation, we performed Ca^2+^ imaging in isolated OLL neurons to avoid cell-to-cell communication. Remarkably, mechanical stimulation induced Ca^2+^ elevation in the isolated neurons with a displacement of 3 μm (Fig. 2E, F), further supporting the hypothesis.

To explore the molecular basis of the mechanosensation of OLL neurons, we further examined their touch-induced Ca^2+^ responses in mechanotransduction-related candidate mutants. So far, DEG/ENaC channels such as MEC-4, MEC-10, and DEG-1, the transient receptor potential (TRP) channel protein TRP-4, and the transmembrane channel-like protein 1 (TMC-1) have been identified as mechano-gated channels in *C. elegans* [30–33]*.* Given that all these proteins as well as the mechano-gated Piezo channels are cation channels [31, 32, 34–36], we investigated whether extracellular Na^+^ might be responsible for the mechanotransduction of OLL neurons. We performed Ca^2+^ imaging by replacing NaCl with NMDG-Cl in the bath. Remarkably, 20 μm displacement-induced Ca^2+^ responses in OLL were largely reduced in the Na^+^-free bath than that in normal solution (Fig. 2E, F). These results suggest that cation channels mediate the mechanosensation of OLL neurons. Since the DEG/ENaC protein MEC-4 is known as a mechanoreceptor which is exclusively expressed in gentle touch neurons such as ALM and PLM [37, 38], we then tested the touch-induced Ca^2+^ transients of OLL neurons in *deg-1* and *mec-10* mutants. Both mutant worms displayed no significant difference in the nose touch-evoked Ca^2+^ response in OLL neurons from that of the wild-type (Fig. 2C, D). Nevertheless, the *deg-1* and *mec-10* mutants showed modest inhibition of the touch-induced Ca^2+^ responses. Thus, our data do not rule out the possibility that DEG-1 and MEC-10 may be involved in the mechanosensation of OLL neurons. Overall, these findings suggest that OLL neurons detect mechanical stimulation through cation channels.

### Mechanotransduction of OLL Neurons Might be Carried by a Novel Sodium Conductance Channel

**Fig. 3:**
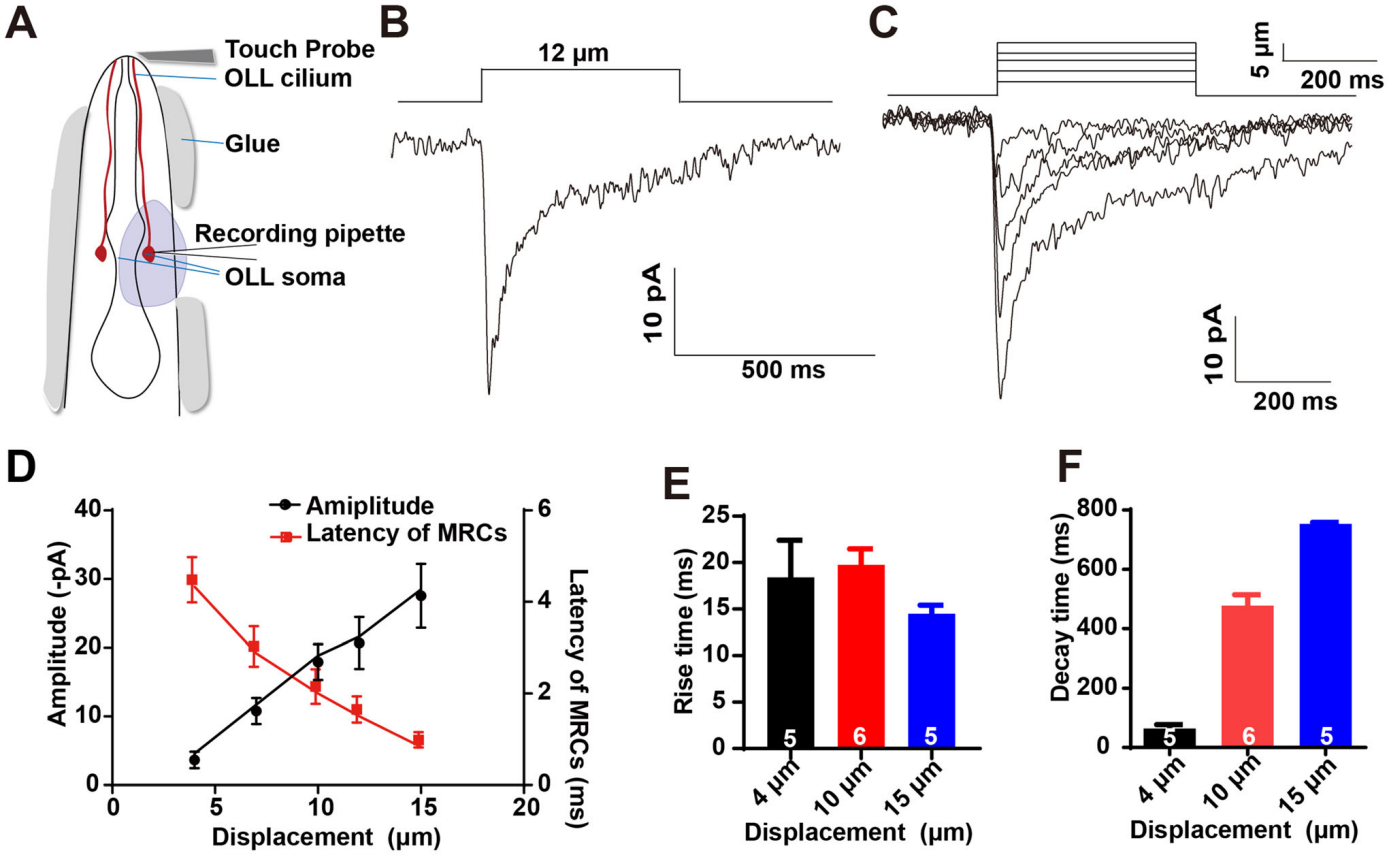
Recordings of mechanoreceptor current (MRC) in OLL neurons *in situ.* **A** Schematic of MRC recording in OLL neurons from a dissected worm. A glass probe (about 10 μm in diameter) driven by a Piezo actuator delivers mechanical stimulation to the cilium of OLL neurons. **B** Representative MRCs in OLL neurons respond to mechanical stimulation with a displacement of 12 μm. Cell membrane potential is voltage-clamped at –70 mV. **C** Sample traces of MRCs in OLL neurons recorded in response to 4 μm, 7 μm, 10 μm, 12 μm, and 15 μm displacement. Cell membrane potential is voltage-clamped at –70 mV. **D** The amplitude and latency of MRCs in OLL neurons are dependent on the strength of stimulation. Note that the latency is close to 1 ms when the displacement is 15 μm. Cell membrane potential is voltage-clamped at –70 mV. The *n* values for the 4 μm, 7 μm, 10 μm, 12 μm, and 15 μm displacement groups are 8, 8, 7, 4, and 6, respectively. **E** The 10%–90% rise time of MRCs in OLL neurons. Note that the rise time is <25 ms. The cell membrane potential is voltage-clamped at –70 mV. The *n* values for the 4 μm, 10 μm, and 15 μm displacement groups are 5, 6, and 5, respectively. **F** The 10%–90% decay time of MRCs in OLL neurons. Note that the decay time is <1 s. The cell membrane potential is voltage-clamped at –70 mV. The *n* values for the 4 μm, 10 μm, and 15 μm displacement groups are 5, 6, and 5, respectively. Error bars indicate SEM.

If OLL neurons do function as mechanoreceptors, a mechanoreceptor current (MRC) responding to mechanical stimulation should be detected by whole-cell patch clamp recording in intact worms [31]. We then performed patch-clamp recording in OLL neurons. Briefly, the cell body was carefully exposed to a recording pipette by micro-dissection while keeping the nose tip in perfect condition (Fig. 3A). Strikingly, mechanical force evoked an MRC (Fig. 3B–D). The 10–90% rise time and the 10–90% decay time of the MRC were <25 ms and 1 s, respectively (Fig. 3E, F). Thus, the MRC was fast and transient. It should be noted that the nose touch-evoked MRC had a very short latency (1.5 ± 0.3 ms, 12 μm displacement, *n* = 4). As the fastest known intracellular second messenger signaling takes more than 5 ms [31, 39–41], it is highly improbable that a second messenger indirectly activates MRCs in OLL neurons. Moreover, we found that the amplitude and latency of MRCs was dependent on the strength of mechanical stimulation (Fig. 3C, D). When a displacement of 15 μm was applied, the MRC latency approached 1 ms (0.8 ± 0.2 ms, *n* = 6). Besides, a displacement of 4 μm was able to evoke a small MRC (Fig. 3C, D). The MRC threshold was much lower than that of mechano-evoked Ca^2+^ transients (Figs. 2A, B; 3C, D), perhaps because the depolarization induced by a small MRC was insufficient to open the voltage-gated Ca^2+^ channels.

**Fig. 4:**
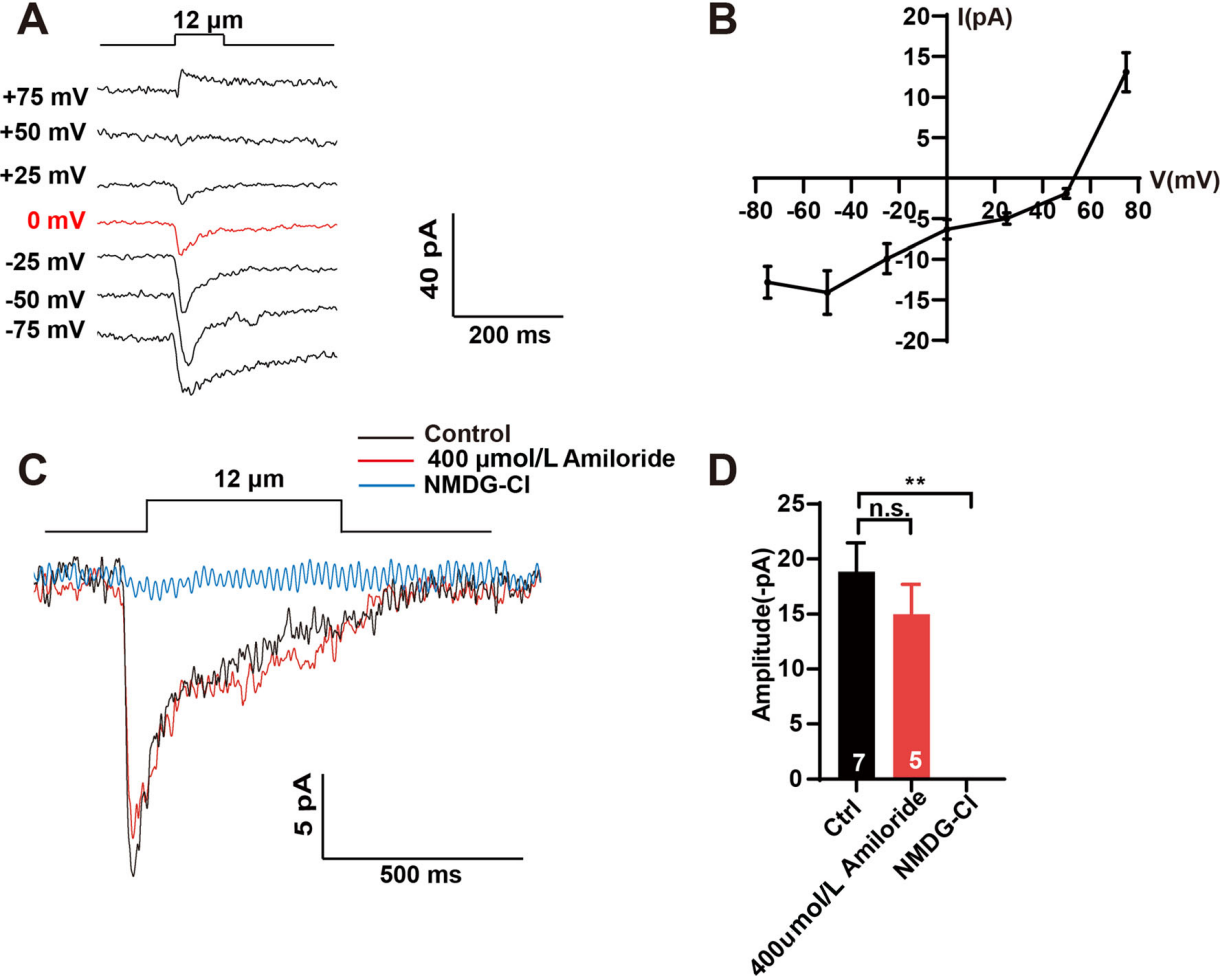
A novel Na^+^ channel mediates mechanotransduction in OLL neurons. **A** Sample traces of MRCs in an OLL neuron recorded with Cs-gluconate intracellular solution and NaCl extracellular solution. Cell membrane potentials are voltage-clamped at +75 mV, +50 mV, +25 mV, 0 mV, –25 mV, –50 mV, and –75 mV. **B** Current-voltage relationships (I–V curves) of MRCs in OLL neurons. Note that the reversal potential is ~53 mV, which is similar to that of Na^+^-selective channels. The *n* values for the +75 mV, +50 mV, +25 mV, 0 mV, –25 mV, –50 mV, and –75 mV voltage-clamped groups were 3, 8, 8, 8, 9, 9, and 9, respectively. **C, D** Sample traces (**C**) and quantification (**D**) of the MRCs in OLL neurons upon 400 μmol/L amiloride treatment and in Na^+^-free bath solution (NMDG-Cl). The *n* values of the control group, the 400 μmol/L amiloride treatment group, and the NMDG-Cl solution group were 3, 7, and 5, respectively. Error bars indicate SEM; n.s., not significant, *P* > 0.05; ** *P* < 0.01.

To further test the biophysical properties of the MRC in OLL neurons, we recorded the current-voltage relationships (I-V curves) of MRCs. The reversal potential of the MRC was 53 mV, which is similar to the reversal potential of the Na^+^ channel conductance but distinct from that of mechano-gated non-selective cation channels such as TRP-4, TMC, and Piezo [31, 32, 34, 35, 42] (Fig. 4A, B). Furthermore, the MRC induced by nose touch with 12 μm displacement was abolished in the Na^+^-deprived bath (Fig. 4C, D). According to previous reports, all known mechano-gated Na^+^ channels belong to the DEG/ENaC family of proteins, which can be effectively blocked by amiloride [31, 38, 40]. However, upon treatment with 400 μmol/L amiloride, the MRCs in OLL neurons were indistinguishable from those in control worms, suggesting that the ENaC channel is not the mechanoreceptor channel in OLL neurons (Fig. 4C, D). These results support the conclusion that the MRCs in OLL neurons are carried by a novel Na^+^ conductance channel.

### OLL Neurons Respond to Cold Temperatures but not Via TRPA-1 Channels

**Fig. 5:**
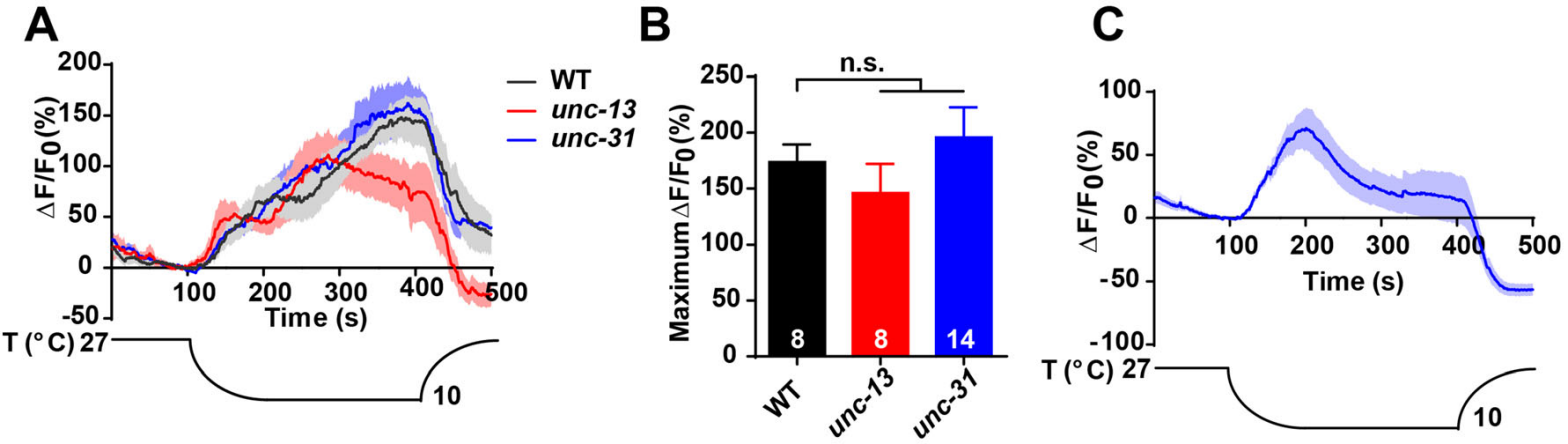
OLL neurons cell-autonomously sense cold. **A, B** Ca^2+^ responses (**A**) and maximum ΔF/F0 changes (**B**) induced by cold stimulation in OLL neurons of wild type (*n* = 8), *unc-13* mutants (*n* = 8), and *unc-31* mutants (*n* = 14). (**C**) Cold stimulation-evoked Ca^2+^ responses in isolated OLL neurons. *N* = 9. Error bars indicate SEM; n.s., not significant.

We next explored the molecular mechanism of responses in OLL neurons activated by cold stimulation. First, we investigated whether OLL neurons can sense cold stimulation cell-autonomously. The cold-evoked Ca^2+^ transients in *unc-13* and *unc-31* mutants were normal compared to the wild-type (Fig. 5A, B), suggesting that OLL neurons act as primary sensory neurons that can sense cold stimulation cell-autonomously. We then performed Ca^2+^ imaging in isolated OLL neurons to confirm that they directly respond to cold stimulation. Interestingly, the isolated OLL neurons responded to cold stimulation, supporting the hypothesis that OLL neurons mediate cold sensation in a cell-autonomous manner (Fig. 5C).

**Fig. 6:**
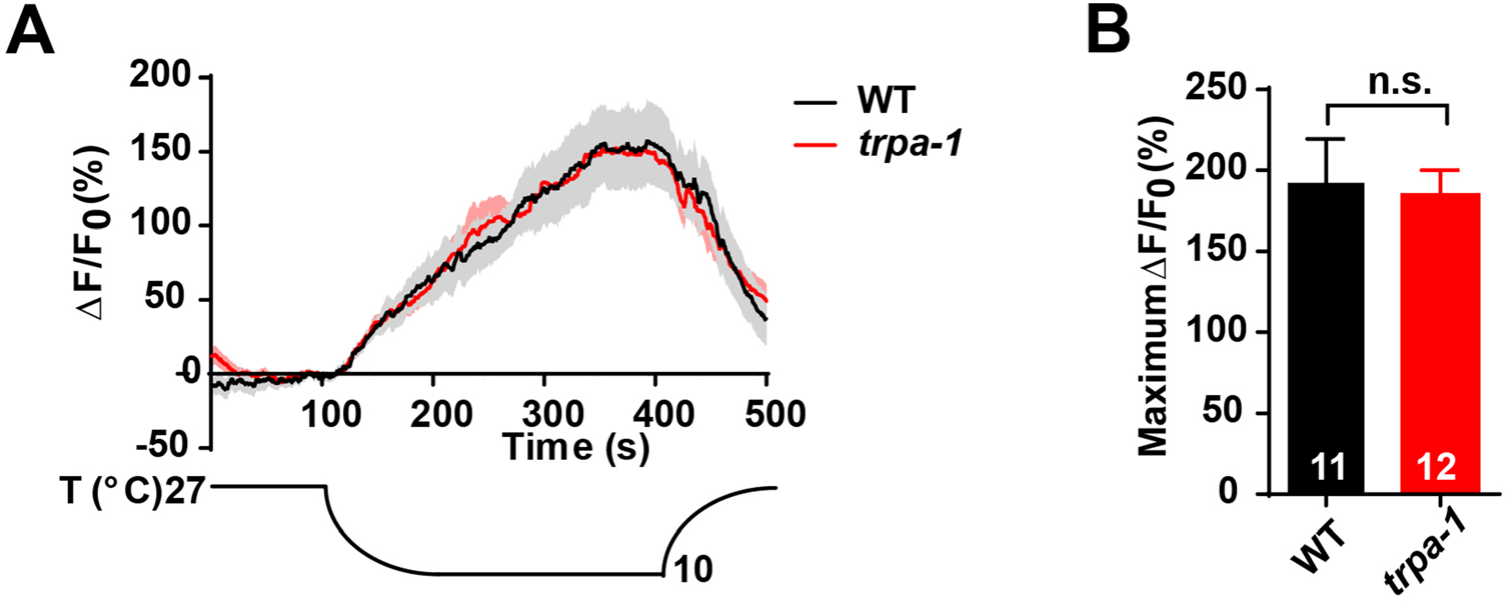
OLL neurons sense cold in a TRPA-1-independent manner. **A, B** Ca^2+^ responses (**A**) and maximum ΔF/F_0_ changes (**B**) induced by cold stimulation in OLL neurons of wild type (*n* = 11), and *trpa-1* mutants (*n* = 12). Error bars indicate SEM; n.s., not significant.

Since OLL neurons directly sense cold, cold receptors must be present in them. Three cold receptors – TRPM8, TRPA1, and GLR-3 – have been identified in mammals [13, 16, 43]. TRPM8 does not have evolutionarily-conserved expression in *C. elegans* [44] and GLR-3 does not seem to be expressed in OLL neurons (https://cengen.shinyapps.io/SCeNGEA/). In this case, since TRPA-1 is a known cold-sensitive receptor in *C. elegans* [22, 45], we enquired whether TRPA-1 was involved in the cold sensitivity of OLL neurons. Interestingly, the cold-induced Ca^2+^ transients were unaffected in *trpa-1* mutants compared to wild-type worms (Fig. 6A, B), suggesting that the cold receptor is not TRPA-1. Hence, there are other unknown cold-sensitive receptors of OLL neurons that remain to be revealed.

## Discussion

All animals are endowed with the highly-conserved ability to detect mechanical stimulation in the form of touch, nociception, and proprioception [1]. *C. elegans* was the first model organism in which unbiased forward genetic screening was used to identify the genes contributing to mechanosensation [46, 47]. Over the years, several polymodal sensory neurons have been identified in the nematode. Polymodal sensory neurons are a specific class of sensory neurons that can detect distinct sensory stimuli. In contrast, neurons that only recognize a single stimulus are unimodal sensory neurons [48]. Nociceptive sensory neurons are typically polymodal with remarkable ability to sense a wide variety of aversive stimuli including physical and chemical stimuli, and to transduce them into diverse degrees and modalities of pain signals [49]. The bilateral pair of ASH neurons (amphid neurons with single ciliated endings) sense chemical, mechanical, and osmotic stimuli to mediate backward locomotion for self-defense [10, 48, 50, 51]. The PVD neurons detect noxious cold temperatures, harsh touch, and are involved in proprioception [21, 22]. The PHA/PHB neurons respond to several aversive stimuli, including chemical, mechanical, and osmotic stimuli [10]. Other sensory neurons are under scrutiny to decipher the molecular basis of the sensory processes and the multimodality.

### OLL Neurons are Novel Polymodal Sensory Neurons

Previous studies using the electron microscopy reconstruction found that OLL neurons have a single dendrite. The cilia of OLL neurons traverse through a channel created by socket cells and the end is finally embedded in the amphid subcuticle [8]. Chang *et al.* reported nose touch defects from animals in which the OLL neurons were genetically ablated, suggesting that they are involved in mechanosensation [11]. Nevertheless, the molecular mechanism of this mechanosensation remained elusive. In the present study, using Ca^2+^ imaging and patch-clamp recording, we revealed nose touch-evoked Ca^2+^ increases and currents in OLL neurons in a cell-autonomous manner. Collectively, OLL neurons are involved in mechanosensation. Intriguingly, OLL neurons are also involved in detecting cold stimulation, which has not been reported before. The behaviors of cold temperature perception, including innocuous cool sensation, noxious cold sensation, positive and negative thermotactic behaviors, and temperature-related memory and aging, are quite complicated [1, 16, 45]. Due to the complexity of this temperature-coding scheme, it is often difficult to identify the temperature behavioral output for a specific neuron. At this stage, we have not observed any obvious behavioral output of OLL neurons in response to cold stimulation. Future studies are needed to reveal these details. Nevertheless, our Ca^2+^ imaging experiments conducted both *in vivo* and *in vitro* clearly showed that OLL neurons responded to cold stimulation in a cell-autonomous manner, supporting the conclusion that they are cold-sensing cells. It is thus clear that OLL neurons act as polymodal sensory neurons functioning in mechanotransduction and cold transduction.

### A Novel Sodium-Sensitive Channel in Mechanotransduction

It is generally known that mechanoelectrical transduction (MeT) channels detect mechanical signals and convert them into electrical signals [1]. MeT channels open very quickly and the channel latencies are typically less than 20 ms [31, 41, 52]. To date, members of four classes of membrane proteins that are related to mechanotransduction are considered to be pore-forming subunits of MeT channels: TRP, DEG/ENaC, Piezo, and TMC [31, 32, 34, 38, 53, 54]. Several members of the DEG/ENaC family such as DEG-1, MEC-4/MEC-10, DEGT-1, and UNC-8, have been shown to form mechanotransduction channels [21, 38, 40, 52]. However, the roles of putative MeT channels in sensory transduction are complicated and perplexing. TRP-4/TRPN forms the MeT channel in the dopaminergic neurons [31, 55], whereas the OSM-9 TRP channel is dispensable for the mechanotransduction by ASH neurons, even though it is essential for ASH-mediated avoidance behaviors in response to nose touch, osmotic, and chemical stimuli [52, 56]. In male ray neurons, OSM-9 modulates the kinetics of Ca^2+^ variations in response to mechanical stimulation [18]. These results support the idea that OSM-9 functions as a modulator but not as the primary MeT channel in mechanosensation.

In this study, we recorded robust, rapidly-adapting amiloride-insensitive MRCs in OLL neurons activated by mechanical force. The MeT channels in these neurons selectively permeated Na^+^ ions, according to the reverse potential of MRCs which is similar to that for Na^+^ channel conductance. TRP-4, TMC, and Piezo channels are all non-selective cation channels involved in mechanosensation, while DEG/ENaC are known to be amiloride-sensitive. As such, we ruled out TRP-4, TMC, Piezo, and ENaC channels as primary MeT channels in OLL neurons [1]. Therefore, the MeT channel of OLL neurons is an unreported mechanotransduction channel that is Na^+^-selective but not amiloride-insensitive. The molecular mechanisms by which OLL neurons function in mechanotransduction remain elusive. Thus, further studies are necessary to uncover this new mechanotransduction channel.

### An As-Yet-Unknown Cold-Sensing Receptor Exists in OLL Neurons

The ability to sense ambient temperature is an indispensable trait for animal survival [1]. Cold temperatures, especially extremely low temperatures, threaten survival, causing severe damage to tissues and provoke cold pain sensation as warning signals [57]. A chain of temperature-sensing mechanisms have been reported to detect cold temperatures to alter behavior and physiology appropriately for survival [58]. Several cold receptors are present in cold-sensitive neurons or tissues [58]. The nematode *C. elegans*, which survives over a relatively wide temperature range from 12°C to 26°C, provides a useful platform for studying the neuro-molecular basis of temperature sensation, particularly cold [59]. So far, only three cold receptors have been identified: TRPM8, TRPA1, and GLR-3. TRPM8 participates in cooling sensation with an activation threshold at ~26°C. The TRPM8 cold receptor has been confirmed both *in vivo* and *in vitro* in mammals [43, 60, 61]. TRPA1 is ubiquitous and relatively conservative in its function [62]. In *C .elegans*, TRPA1 is responsible for cold sensation in PVD neurons and the intestine [22, 45]. However, OLL neurons exhibited robust Ca^2+^ transients evoked by cold stimuli with *trpa-1* mutation; thus TRPA1 did not contribute to the cold transduction in OLL neurons.

A recent study identified GLR-3 as a metabotropic cold receptor whose activation threshold is about 20°C [16]. GLR-3 is reported to be in ASER (the amphid neuron, single (AsE) ciliated ending, right side) to function in cold sensation and mediate cold-avoidance behavior. The *glr-3* gene is expressed in the ASER sensory neuron, RIA interneurons, and the intestine, as well as some other neurons, but not in OLL neurons (https://cengen.shinyapps.io/SCeNGEA/) [16]. Given that TRPM8 does not have evolutionarily conserved expression in *C. elegans*, it remains unclear which cold receptor exists in OLL neurons. Prospective studies are required to identify the cold receptor as well as the detailed transduction mechanisms.

In summary, we demonstrated that polymodal OLL neurons mediate both mechanotransduction and cold transduction cell-autonomously. We ruled out the DEG/ENaC family proteins, TRP-4, TMC, and Piezo/Pezo-1 proteins in the mechanotransduction of OLL neurons, however, we provided fundamental insights into a novel mechano-sensitive Na^+^ channel. Another interesting observation was that OLL neurons sensed low temperatures through an unknown cold receptor. Although three cold receptors have been identified, this unexpected cold receptor instigates new avenues for cold-sensing mechanisms. With further investigations, we expect that our study will contribute to advancing the molecular understanding of polymodal sensory neurons.
